# The response to stressors in adulthood depends on the interaction between prenatal exposure to glucocorticoids and environmental context

**DOI:** 10.1038/s41598-023-33447-x

**Published:** 2023-04-15

**Authors:** Ariana D. Majer, Ryan T. Paitz, Gianna M. Tricola, Jack E. Geduldig, Hannah P. Litwa, Jenna L. Farmer, Brenna R. Prevelige, Elyse K. McMahon, Taylor McNeely, Zach R. Sisson, Brian J. Frenz, Alexis D. Ziur, Emily J. Clay, Brad D. Eames, Shannon E. McCollum, Mark F. Haussmann

**Affiliations:** 1 Department of Biology, Bucknell University, Lewisburg, PA 17837 USA; 2 School of Biological Sciences, Illinois State University, Normal, IL 61790 USA

**Keywords:** Ecology, Evolution, Physiology

## Abstract

Maternal stress during reproduction can influence how offspring respond to stress later in life. Greater lifetime exposure to glucocorticoid hormones released during stress is linked to greater risks of behavioral disorders, disease susceptibility, and mortality. The immense variation in individual’s stress responses is explained, in part, by prenatal glucocorticoid exposure. To explore the long-term effects of embryonic glucocorticoid exposure, we injected Japanese quail (*Coturnix japonica*) eggs with corticosterone. We characterized the endocrine stress response in offspring and measured experienced aggression at three different ages. We found that prenatal glucocorticoid exposure affected (1) the speed at which the stress response was terminated suggesting dysregulated negative feedback, (2) baseline corticosterone levels in a manner dependent on current environmental conditions with higher levels of experienced aggression associated with higher levels of baseline corticosterone, (3) the magnitude of an acute stress response based on baseline concentrations. We finish by proposing a framework that can be used to test these findings in future work. Overall, our findings suggest that the potential adaptive nature of prenatal glucocorticoid exposure is likely dependent on environmental context and may also be tempered by the negative effects of longer exposure to glucocorticoids each time an animal faces a stressor.

## Introduction

Change is a persistent feature of most environments, and organismal survival requires physiological and behavioral flexibility to cope with environmental perturbations. That flexibility is conferred by an organism’s phenotype, which is largely dependent on their genetic background. However, the phenotype can also be shaped by parental effects^1,2^. Specifically, embryonic exposure to maternal factors, such as hormones and antibodies, can act as a mechanism to match the offspring’s phenotype to the prevailing selective conditions experienced by the mother^1,3,4^. These prenatal maternal effects are major contributors to both phenotypic variation and fitness differences between individuals^1^. Determining how maternal effects influence an organism’s preparation for their future environment is therefore a key aspect of the contemporary research agenda in evolutionary biology.

When faced with environmental threats, individuals activate an evolutionarily conserved suite of stress responses to cope with the challenges. Activation of a stress response induces transient physiological and behavioral changes that maximize survival and can be turned off once the challenge has subsided. In vertebrates, the endocrine stress response is a multifaceted yet highly conserved process that is critical in allowing individuals to restore homeostasis after environmental perturbations^5^. One major pathway of the endocrine stress response is the hypothalamic–pituitary–adrenal (HPA) axis. In brief, perceived stressors activate the hypothalamus to secrete corticotropin-releasing hormone (CRH), which stimulates the pituitary gland to release adrenocorticotropic hormone (ACTH). This, in turn, causes the release of glucocorticoids from the adrenal cortex^6^. Glucocorticoid release promotes survival by inhibiting unnecessary physiological functions, regulating behaviors that control energy intake and expenditure, and mobilizing energy stores that help the animal to recover from the stress^5,7,8^. Upon termination of the stressor, homeostasis is typically restored, returning glucocorticoids to baseline levels.

The acute stress response can be characterized through three measures: baseline, stress-induced, and recovery glucocorticoid levels^9,10^. Baseline glucocorticoids can be measured directly before an acute stressor and are linked to an individual’s current energetic state^11^, with higher glucocorticoid levels being indicative of more frequent environmental challenges^12^. Stress-induced glucocorticoids are a measure of circulating glucocorticoids during a stressor, and they can provide information on glucocorticoid reactivity in response to a stressor. In general, a larger stress-induced increase in glucocorticoids indicates a larger investment of energy toward self-maintenance and survival, whereas a smaller increase in glucocorticoids suggests an individual is instead allocating energy to reproduction and other non-self behaviors^13^. Together, baseline and stress-induced glucocorticoid measures provide information on the hormonal control of energy mobilization, with baseline reflecting an organism’s ability to cope with longer-term, predictable seasonal demands^14^ and stress-induced reflecting their ability to mount short-term, plastic responses when faced with environmental uncertainty^15^. The third measure, recovery glucocorticoids, is a measure of an individual’s ability to turn off the stress response once the stressor has ended, as it captures glucocorticoid levels as they decrease toward baseline. Recovery glucocorticoid levels are dependent on the strength of the negative feedback systems of the individual’s HPA axis, with recovery glucocorticoid levels more similar to baseline levels indicating stronger negative feedback^11^. A quicker return to baseline is generally considered more beneficial to the individual because it decreases the duration of glucocorticoid exposure, and prolonged exposure to glucocorticoids is associated with increased levels of oxidative stress^10,16^, cardiovascular disease^17^, depression^18^, and mortality risk^19^.

Because variation in the acute stress response is tied to variable health outcomes, identifying the factors contributing to phenotypic variation in the stress response is of great interest. One possible contributing factor is maternal effects. Maternal effects are ubiquitous in nature^20^, so in addition to how they may affect the stress response, their ecological and evolutionary implications are currently the subject of intense research. Recent reviews have emphasized the need for more empirical work to better quantify how variation in the maternal environment translates to phenotypic changes in the offspring, and how these changes behave in different environmental contexts^20,21^. Maternal stress provides a verdant field for these inquiries, as glucocorticoids play a significant role in the management of homeostatic energy balance and have thus been proposed as a major mechanistic translator that provides offspring with information about the maternal environment^22^. The information provided by maternal glucocorticoids is therefore thought to have a direct impact on offspring success in their postnatal and future environments^22^. In order to investigate this relationship, we manipulated yolk glucocorticoids in Japanese quail (*Coturnix japonica*) to study the influence of maternal glucocorticoids on offspring stress responses over the course of their lifespan and in variable environments. In doing so, we sought to address two research questions:

- How does embryonic exposure to glucocorticoids (an indicator of the maternal environment) alter the function of the offspring HPA axis throughout the offspring’s lifespan?
- Does embryonic exposure to glucocorticoids interact with the level of social stress experienced by an individual in their postnatal environment to affect their acute stress response?

The first question is based on the knowledge that elevated maternal glucocorticoids can permanently modify the development and subsequent function of the HPA axis in offspring^6,9,16,23,24^. While there is evidence that developing offspring can regulate their exposure to maternal glucocorticoids through the extraembryonic membranes^25–27^, even small amounts of glucocorticoids that reach the embryo can result in altered programming^23,24,28^. Presently, there is an appreciation that within-individual variation in endocrine traits has been understudied^29^, and maternal glucocorticoid exposure may be an important source of that variation. Furthermore, studies have shown that maternal exposure can alter baseline and stress-induced glucocorticoid levels, as well as the ability for an individual to turn off an acute stress response^16,30–33^. These changes may in part be due to altered expression of glucocorticoid receptors in the hippocampi of prenatally stressed offspring^34–36^. While maternal stress effects have mostly been considered maladaptive to offspring^16,17,31,37^, there is also research that suggests maternal stress effects can be adaptive, serving to prepare offspring that grow up in similarly stressful environments^38–40^.

The nature of the benefit or consequence of maternal stress to offspring leads to the second question, which reflects that the effects of past maternal stress can only be viewed through the lens of current environmental conditions that are constantly subject to change. While specific prenatally programmed stress responses might be adaptive in some postnatal environments, they can just as easily become maladaptive as the environment changes^29^. Furthermore, while the benefits of producing an acute stress response in the face of a stressor are clear, there are consequences to producing acute stress responses too frequently, such as increased disease susceptibility and decreased longevity^5,9,41^. In social species, group living can buffer environmental challenges, such as through increased protection from predators or easier access to potential mates, both of which provide an evolutionary advantage^42–45^. However, group dynamics can also be a significant source of stress through agonistic encounters^42,46–48^. While Japanese quail form dominance hierarchies, the hierarchies are notably unstable and change through time depending on resource availability, season, and dynamic group relationships^49^. We capitalized on this socially unstable system to determine how experimental embryonic glucocorticoid manipulation interacts with the current natural social environment to affect acute physiological stress responses.

## Methods

All procedures were conducted with approval from the Bucknell University Institutional Animal Care and Use Committee and the reporting. All procedures were in compliance with recommended ARRIVE guidelines^50^.

### Study species

A breeding colony of Japanese quail (*Coturnix japonica*) from a feral line originally captured on the Big Island of Hawaii in 1980 was maintained at Bucknell University as previously described^10^. Because the birds were wild until recently, they have faced different natural selection pressures and have undergone less intensive artificial selection than domestic breeds of *Coturnix japonica*^51^. In our colony, the birds have a mean lifespan 2.1 ± 1.08 (sd) years. The birds were housed in a 10 × 10-foot pen and maintained on a light–dark cycle that mimicked the outdoor ambient light cycle in Lewisburg, Pennsylvania. Throughout the entire study, all birds received ad libitum access to food (Sporting Bird Starter and Sporting Bird Flight Developer, Southern States, USA) and water.

### Injection, incubation, and hatching

**Figure 1:**
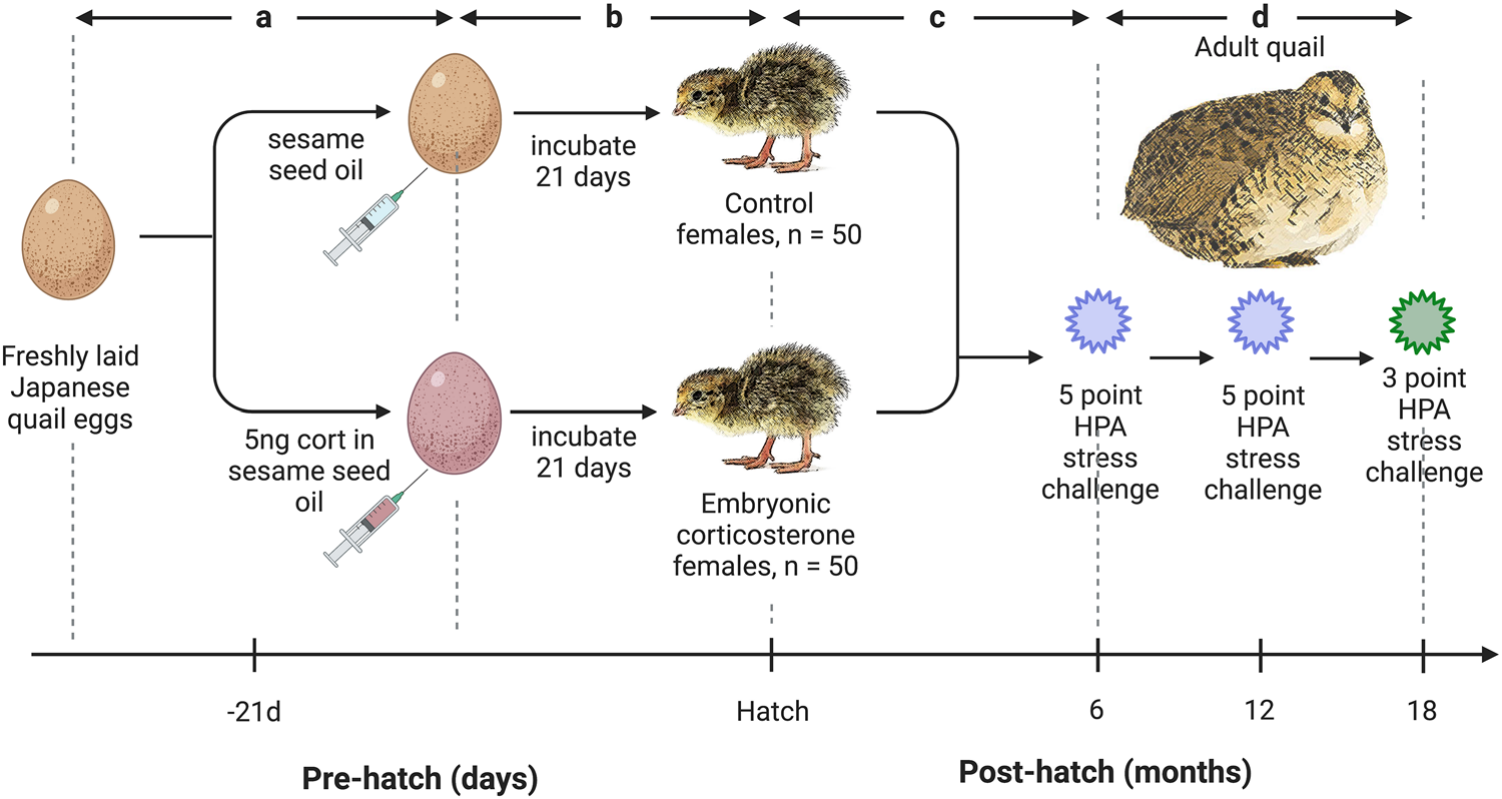
Experimental design timeline. (**A**) Freshly laid eggs were injected with sesame oil (control) or corticosterone in sesame oil within 24 h of being laid. (**B**) Injected eggs were incubated for 21 days, and after hatch chicks were sexed and a total of 100 female birds were included in the experiment (n = 50 each group). (**C**) Birds remained in the brooder for 4 weeks and then were transferred to pens. (**D**) HPA acute stress series were repeated throughout adult life, with 5-point challenges occurring at 6 and 12 months of age, and a 3-point challenge occurring at 18 months of age.

Freshly laid eggs (ranging from 18 to 24 eggs per day) were collected daily between May 17 and May 26 of 2016 from the breeding population (Fig. 1a). Within 24 h of laying, the eggs were randomly sorted into one of two groups and injected with either a 5 µL bolus of sesame oil (control group) or a 5 µL bolus of sesame oil containing 5 ng of corticosterone (Sigma), the primary avian glucocorticoid (embryonic corticosterone group, going forward the CORT-treated birds; Fig. 1a). This corticosterone concentration was chosen because it was within the physiological range found in twenty eggs of this species in our laboratory (unpublished data) housed under normal recommended conditions^52^. Additionally, this is a high enough concentration to result in some corticosterone reaching the embryo without first being metabolized by enzymes in the extraembryonic membranes^27^. The oil and corticosterone solutions were injected into the yolk using a Hamilton syringe as previously described^27^. Injection order of the eggs was chosen randomly, and injections alternated between corticosterone and vehicle solution.

After injection, each egg was placed in a marked but randomized separate compartment of a GQF 1502 Digital Sportsman incubator (GQF Manufacturing Co., Savannah, GA, USA) to easily track its identity and treatment. The incubator was maintained at 37.8°C and 50–65% humidity while being turned every 2 h (Fig. 1b). On day 15 of incubation, the cohort was transferred to a GQF 1550 Digital Hatcher (1550 Cabinet Model, GQF Manufacturing Co., Savannah, GA, USA) kept at 98 °C and 85% humidity. Starting at day 18 of incubation, the cohort was checked every 12 h for hatches. After hatching, the birds were weighed, marked with numbered leg bands, and housed in a Brower brooder maintained at 38 °C and decreased at a rate of 2.5 °C per week until 24 °C was reached. At 14 days of age, blood samples were collected from the alar vein and sex was determined via molecular sexing assay (see below). Due to facility space restrictions and concerns regarding statistical power, only the females in both treatment groups remained in the study, and birds were added until we reached n = 50 per treatment group (Fig. 1b). Birds remained in the brooder until 4 weeks of age, when they were transferred to 3.5 × 9-foot pens (n = 10 control and n = 10 CORT-treated per pen; Fig. 1c). Birds were weighed every two weeks using a top loading balance to monitor their health.

In addition to the one hundred study birds, twenty birds (n = 10 per treatment group) were held in reserve and housed in a separate pen from the study birds. In the case of death of a study bird prior to the first stress series at 6 months of age, a reserve bird from the corresponding treatment group was added to the study pen that lost a bird as a replacement. This occurred four times during the study: twice in the control group and twice in the CORT-treated group.

### HPA axis function

At 6, 12, and 18 months of age, all birds were subjected to an acute stress series following methods similar to those previously established in our laboratory^16^ in order to assess HPA axis activity (Fig. 1d). For the stress series conducted at 6 and 12 months of age, five blood samples (approximately 75 µL each) were collected from the alar vein representing five different aspects of the vertebrate stress response (baseline, stress-induced, dexamethasone-suppression, adrenal capacity, and recovery; Fig. 1d). At 18 months of age, we conducted a modified three time point stress series (baseline, stress-induced, and recovery; Fig. 1d) in order to better understand an individual’s natural ability to turn off the stress response following termination of the stressor.

All stress series began at noon on Friday. The five-point series lasted for approximately 3 h, while the three-point series lasted approximately 1 h. Twenty birds (ten in each treatment) were assessed on five consecutive Fridays. On a given Friday, we performed the stress series on two groups of ten birds. The stress series start time was staggered by 10 min between the two groups, and there were six to eight experimenters simultaneously collecting blood samples. Each group of ten birds had an even number of control and CORT-treated birds. A partition in the animal rooms allowed for us to enter the room with the first group of ten birds without disturbing the second group of ten birds.

#### Five-point stress series at 6 and 12 months

##### Baseline

All blood samples were collected within 1.5 ± 0.8 min of entry into the room housing the birds to determine the individual’s baseline level of circulating glucocorticoids. Previous studies have shown that, in birds, it typically takes between three and five minutes to observe a measurable increase in glucocorticoid levels^11,53^. However, because some studies^54,55^ report that individuals differ in the timing of initial activation of the stress response, we determined whether the time between disturbing the birds and collecting baseline blood samples was related to baseline corticosterone levels, but there was no significant relationship between them (R^2^ = 0.0002, F_1,299_ = 0.06, P = 0.8). This suggests that our baseline blood samples were not influenced by the time it took to collect those samples.

##### Stress-induced

After taking the baseline sample, birds were placed in an opaque, breathable cotton bag and left alone. This functioned as a mild acute stressor. At 30 min following initial entry into the room, the stress-induced blood sample was taken.

##### Dexamethasone suppression

Immediately following collection of the stress-induced blood sample, birds were injected subcutaneously with the synthetic glucocorticoid dexamethasone (1000 µg/kg in 50 µL injection volume) and then placed back into the cloth bag. Twenty minutes later, and 50 min from initial entry into the room, the dexamethasone-suppression blood sample was collected. The dexamethasone injection allowed us to assess the ability of the negative feedback system to suppress endogenous glucocorticoid release in response to the acute stressor^11,56–58^. The concentration of the dexamethasone injection was validated in our laboratory (Supplementary Fig. S1).

##### Adrenal capacity

Immediately following collection of the dexamethasone suppression blood sample, birds were injected subcutaneously with ACTH (100 IU/kg in 50 µL injection volume) and then placed back into the cloth bag. One hour later, and 110 min from initial entry into the room, the adrenal capacity blood sample was collected. Since release of ACTH by the pituitary gland is the rate-limiting step in the regulation of glucocorticoid secretion^9^, injection of ACTH provides insight into the maximal amount of glucocorticoids each individual was capable of releasing. The concentration of ACTH injected was validated in our laboratory (Supplementary Fig. S2).

##### Recovery

After the adrenal capacity blood sample, birds were returned to their home pens to allow them to recover from the stressor. One hour later, and 170 min from initial entry into the room, the recovery blood sample was collected to assess an individual’s progression back towards baseline upon termination of the stressor in order to determine the individual’s ability to turn off the stress response.

#### Three-point stress series at 18 months

At 18 months of age, an alternative HPA challenge was used to better understand an individual’s natural ability to turn off the stress response following termination of the stressor, meaning these birds were not exposed to hormone injections. The baseline sample (t = 0) and stress induced samples (t = 30) were the same as previously described. However, after the stress-induced sample, birds were returned to their home pens to allow them to recover naturally from the acute bag restraint stressor. Twenty minutes later, and 50 min from initial entry into the room, we collected the recovery blood sample to assess an individual’s natural progression back towards baseline.

All blood samples were kept on ice until completion of the stress series, at which point they were centrifuged at 3500 rpm for 5 min at 7 °C. Plasma was removed and stored at − 20 °C, and the red blood cells were resuspended in cryoprotectant buffer (10% dimethyl sulfoxide, 90% newborn bovine serum) and stored at − 80 °C.

### Molecular sex determination

To identify the sex of the birds, genomic DNA was extracted from blood samples taken at two weeks of age using a modified version of the protocol developed by Griffiths et al.^59^. We amplified DNA using standard polymerase chain reaction (PCR) conditions using the P8 ⁄ P2 primer set to determine the sex of each bird based on the chromo-helicase-binding domain (CHD) gene in poultry^60^. Sex was assigned based on the absence (male:ZZ) or presence (female:ZW) of the band for the W chromosome.

### Aggression quantification

Dominance hierarchy instability and frequent challenges from lower-ranking individuals to higher-ranking individuals is common in Japanese quail^49^. During these challenges, both male and female quail frequently mount and peck at the back of other birds^61,62^. While these behaviors are normal in the social groups of Japanese quail, they can create a socially stressful environment, which can increase circulating levels of corticosterone^63–65^. The feral line of Japanese quail we studied have undergone far less domestication in comparison to most Japanese quail, and show significantly higher levels of aggression in both sexes compared to domestic Japanese quail^51^. We calculated experienced aggression for each quail based on weekly observations of behavior including chasing and pecking, loss of back and head feathers from mounting, and superficial head injuries.

Beginning 5 weeks prior to each of the three stress series, we calculated presence or absence of new signs of aggression experienced for each bird during the past week. Birds were considered to have experienced aggression if we observed new feather loss or fresh wounds consistent with mounting and pecking of the back and head within the past week. We then calculated the proportion of the five weeks where each bird experienced aggression by summing the number of weeks a bird experienced aggression and dividing by five. The resulting aggression score provided a numerical index for the amount of aggression each quail received. (Experienced aggression scores ranged from 0 to 1.0). Separate aggression scores were calculated for all birds for each of the three stress series (6, 12, and 18 months).

### Corticosterone RIA

We determined plasma corticosterone levels by radioimmunoassay (RIA), based on the protocol of Wingfield and Farner^66^ and validated in our laboratory^10^. Briefly, we extracted corticosterone from plasma diluted with double deionized water using anhydrous diethyl ether. The samples were dried with nitrogen gas and then resuspended in 90% ethanol and stored at 4 °C overnight. Samples were then centrifuged, and the supernatant was dried under nitrogen gas and resuspended in phosphate-buffered saline with gelatin (PBSg).

We ran the samples in a competitive-binding RIA with a corticosterone-specific antibody Corticosterone-3-Carboxymethyloxime:BSAhost:rabbit (MP Biomedicals, Solon, OH, USA) and tritiated corticosterone (2000 cpm, NET 399, New England Nuclear Research Products, Boston, MA, USA). Bound and free corticosterone were separated using dextran coated charcoal. After centrifugation, charcoal was removed and radioactivity was determined using a liquid scintillation counter. Plasma CORT concentrations were determined from comparison against a standard curve. The average detection limit was approximately 0.30 ng/mL. All samples were run in duplicate, which were averaged for analysis, and the intra-assay and inter-assay coefficients of variation were 11.2% and 8.9%, respectively.

### Statistics

We ran statistical analyses using JMP Pro software (v.14.0.0, SAS Institute Inc. 2018, Cary, NC, USA). For all analyses, we performed generalized linear mixed models of restricted maximum likelihood (REML-GLMM). For every model, we checked for homogeneity of variances (Levene’s test), and for normality of residuals (Kolmogorov–Smirnov test). We introduced ‘individual’ as a random factor in each model to control for the non-independence of data due to repeated measurements on the same individuals. For corticosterone analyses, prenatal treatment (two treatments), age (three ages), stress series timepoint (five time points at 6 months and 12 months, and three time points at 18 months), and all interactions were included as fixed effects. We sequentially removed non-significant interactions from the models, starting from the higher order interactions, and repeated the analyses until we obtained a model with only significant terms. We carried out post hoc comparisons using Tukey HSD tests. Corticosterone repeatability was calculated using the within- and between-variance components in a linear mixed effects model (LMM), following the restricted maximum-likelihood method (REML) with bird identity as the grouping random factor^67,68^.

Our results suggested a specific, hypothesized pathway of effects among embryonic corticosterone exposure, experienced aggression, baseline corticosterone, and stress-induced corticosterone. We assessed this causal pathway of effects post-hoc using a path analysis in JMP Pro software (v.14.0.0, SAS Institute Inc. 2018, Cary, NC, USA). Path analysis is a special case of structural equation models (Shipley 2000) that specifies how variables are linked together^69^.

## Results

### Embryonic corticosterone exposure prolongs recovery from an acute stressor at all three ages

**Figure 2:**
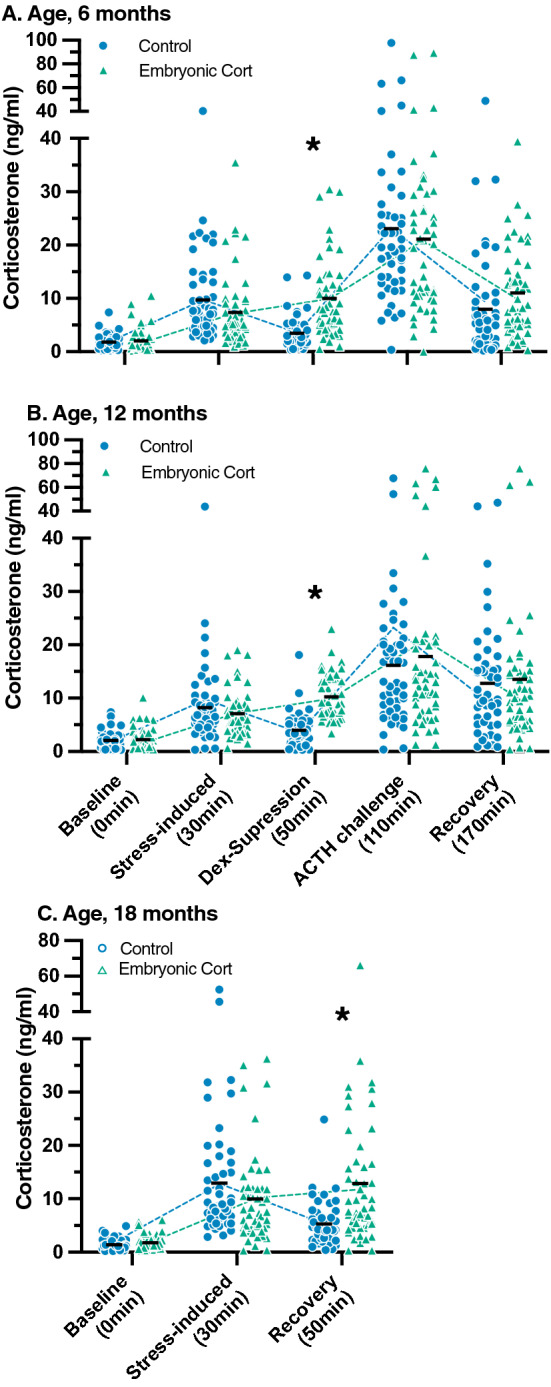
Corticosterone concentrations for each component of the five-point HPA challenge at (**A**) 6 months and (**B**) 12 months, and the reduced three-point HPA challenge at (**C**) 18 months. All control (blue circle) and 5 ng embryonic corticosterone (green triangle) individuals are represented. Black dashes represent group means and are connected by dashed lines for each treatment. Points are jittered for clarity of illustration. Differences between treatments at the same HPA challenge timepoint are marked with an asterisk (Tukey HSD < 0.05). Because the majority of corticosterone values fell between 0 and 40 ng/ml, we split the y-axis at 40 ng/ml to more easily see the values between 0 and 40 ng/ml.

In the five point stress series, there was a significant treatment by stress series time point interaction at both 6 (F_4,396_ = 4.4; p = 0.0017) and 12 months of age (F_4,396_ = 2.6; p = 0.03; Table 1 and Fig. 2a,b). As expected, there was a significant increase in stress-induced corticosterone from baseline levels (all pairwise comparisons; p < 0.03) for both treatments at both ages. However, while corticosterone in the control birds then decreased in the dexamethasone-suppression sample (all pairwise comparisons; p < 0.02), in CORT-treated birds corticosterone remained elevated at the dexamethasone-suppression sample (all pairwise comparisons; p > 0.8, Fig. 2a,b). Regardless of this treatment difference, birds increased their corticosterone levels after challenge with ACTH (p < 0.0009). Also, regardless of treatment, birds decreased circulating corticosterone concentrations between ACTH challenge and the recovery sample at 6 months of age (p < 0.0001), but not at 12 months of age (p = 0.2).

**Table 1:** Results of generalized linear mixed models (GLMM) analyzing the response to treatment (control or 5 ng corticosterone), and stress-series time point (baseline, stress-induced, dexamethasone-suppression, ACTH challenge, and final recovery for 6 and 12 months; and baseline, stress-induced, and recovery for 18 months), and their interaction on corticosterone concentrations. Bold values indicate p < 0.05.

Response variable	Treatment	Stress series time point	Treatment × stress series time point
Corticosterone, 6 months	F_1,99_ = 0.91, p = 0.34	**F**_**4,396**_** = 75.42, p < 0.0001**	**F**_**4,396**_** = 4.42, p = 0.002**
Corticosterone, 12 months	F_1,98_ = 2.37, p = 0.12	**F**_**4,392**_** = 44.13, p < 0.0001**	**F**_**4,392**_** = 2.62, p = 0.03**
Corticosterone, 18 months	F_1,97_ = 1.81, p = 0.18	**F**_**2,94**_** = 58.66, p < 0.0001**	**F**_**2,194**_** = 12.73, p < 0.0001**

At 18 months of age, we conducted the three-point stress series that measured the innate ability of the birds to recover from an acute stressor in the absence of dexamethasone suppression. Once again there was a significant treatment by stress series timepoint interaction (F_4,392_ = 2.6; p = 0.03; Table 1 and Fig. 2c). In both treatments, there was a significant increase in corticosterone from the baseline to the stress-induced sample (all pairwise comparisons; p < 0.0001). Interestingly, while corticosterone in the control birds decreased between the stress-induced sample and the recovery sample (p = 0.04), corticosterone in the CORT-treated birds did not decline between the stress-induced sample and the the recovery sample (p = 0.8).

In addition, while we did find some statistically significant relationships on the repeatability of corticosterone levels (Supplementary Table S1 and Supplementary Fig. S3), and also between age and corticosterone levels across our HPA challenge (Supplementary Table S2), neither corticosterone repeatability nor age showed any consistent patterns (Supplementary Note S1).

### Social aggression increased baseline corticosterone levels in birds exposed to embryonic corticosterone, and baseline corticosterone levels affected stress-induced levels

We consistently observed social aggression in each pen through the duration of our study. As expected, experienced aggression scores were not repeatable, indicating that the birds that experienced the highest levels of aggression at 6 months did not necessarily experience the highest levels of aggression at 12 or 18 months, and vice versa (Supplementary Table S3).

**Figure 3:**
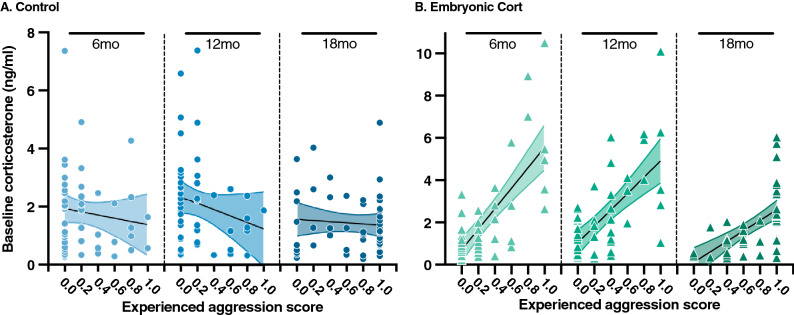
Cumulative experienced aggression score from the five weeks prior to the stress series plotted against baseline corticosterone at 6, 12, and 18 months for (**A**) control birds and (**B**) birds treated with 5 ng embryonic corticosterone. Linear regression lines for each treatment with 95% confidence intervals are plotted for each age.

**Figure 4:**
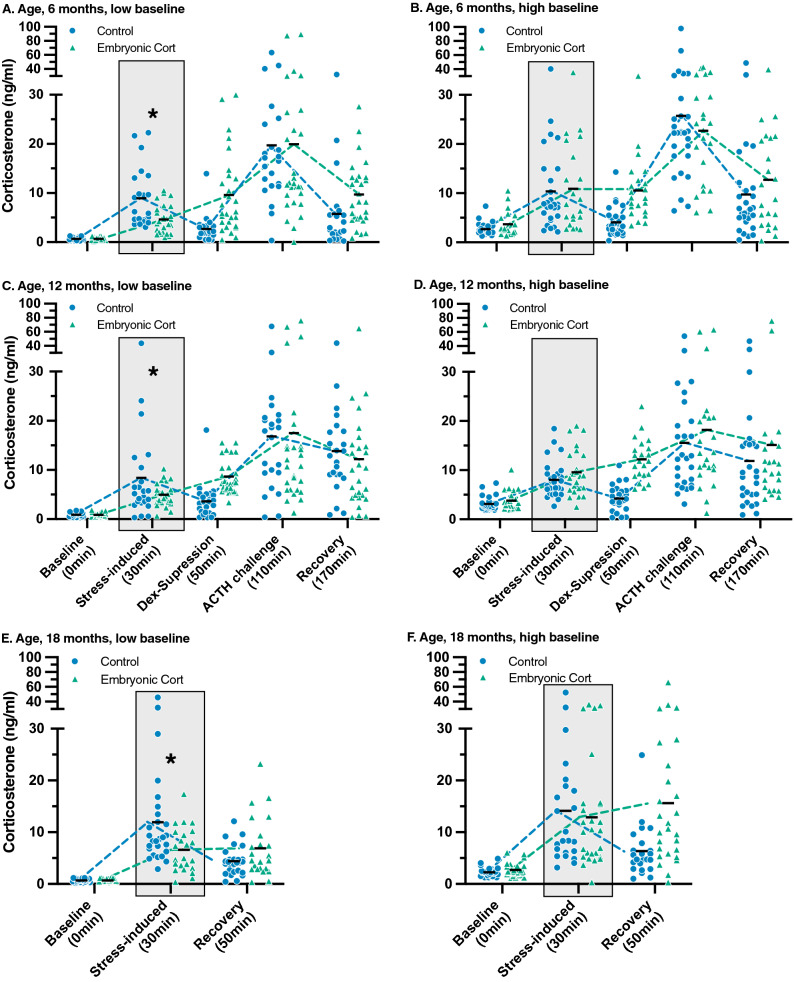
At each age, all birds were divided into those that had baseline corticosterone levels below the population median for that age and those with baseline corticosterone levels above the population median for that age (low vs. high, n = 25 per group). For both low and high baseline groups, corticosterone level is plotted for each component of the five-point HPA challenge at (**A**,**B**) 6 months and (**C**,**D**) 12 months, and the reduced three-point HPA challenge at (**E**,**F**) 18 months respectively. A gray box was placed over the stress-induced timepoint to more easily compare data between the low and high baseline groups and across the three ages. All control (blue circle) and 5 ng embryonic corticosterone (green triangle) individuals are represented. Black dashes represent group means and are connected by dashed lines for each treatment. Points are jittered for clarity of illustration. Significant differences in the effect of baseline by treatment interactions on the different stress challenge parameters are marked with an asterisk (Tukey HSD < 0.05). Because the majority of corticosterone values fell between 0 and 40 ng/ml, we split the y-axis at 40 ng/ml to more easily see the values between 0 and 40 ng/ml.

Interestingly, while baseline corticosterone did not vary with aggression score in control birds (Fig. 3; Table 2), baseline corticosterone did increase with aggression score in CORT-treated birds (Fig. 3; Table 2). This resulted in two distinct patterns of stress responses that were dependent on baseline corticosterone levels (Table 3). To better visualize the two patterns, we divided all birds into those with baseline corticosterone levels below the population median for their age and those with baseline corticosterone levels above the population median for their age (n = 25 per treatment group). This shows that control birds with high baseline corticosterone and CORT-treated birds with high baseline corticosterone had similarly high stress-induced corticosterone (Table 3, Fig. 4b,d,f). However, CORT-treated birds with low baseline levels had significantly lower stress-induced corticosterone levels compared to the control birds with low baseline levels. In other words, the low-baseline CORT-treated birds exhibited a less severe stress response compared to all other birds in the study (Fig. 4a–f).

**Table 2:** Results of generalized linear mixed models (GLMM) analyzing the response to treatment (control or 5 ng corticosterone), aggression score, and their interaction on baseline corticosterone concentrations. Bold values indicate p < 0.05.

Age of Baseline corticosterone	Treatment	Aggression score	Treatment × aggression score
Baseline corticosterone, 6 months	F_1,98_ = 0.4, p = 0.5	**F**_**1,98**_** = 19.1, p < 0.0001**	**F**_**1,99**_** = 30.2, p < 0.0001**
Baseline corticosterone, 12 months	F_1,97_ = 0.001, p = 0.9	**F**_**1,97**_** = 7.7, p = 0.007**	**F**_**1,98**_** = 24.1, p < 0.0001**
Baseline corticosterone, 18 months	F_1,96_ = 2.1, p = 0.1	**F**_**1,98**_** = 15.1, p = 0.0002**	**F**_**1,97**_** = 20.8, p < 0.0001**

**Table 3:** Results of generalized linear mixed models (GLMM) analyzing the response to treatment (control or 5 ng corticosterone), baseline corticosterone group, and their interaction on corticosterone concentrations at different timepoints in the stress series. Bold values indicate p < 0.05.

Response variable	Age	Predictor variables	Results
Stress-induced corticosterone	6 months	**Baseline group**	**F**_**1,100**_** = 12.0, p = 0.0008**
		**Treatment**	**F**_**1,100**_** = 6.4, p = 0.01**
		**Treatment × baseline group**	**F**_**1,100**_** = 11.1, p = 0.001**
Stress-induced corticosterone	12 months	Baseline group	F_1,99_ = 0.7, p = 0.4
		Treatment	F_1,99_ = 3.5, p = 0.06
		**Treatment × baseline group**	**F**_**1,99**_** = 4.7, p = 0.03**
Stress-induced corticosterone	18 months	Baseline group	F_1,98_ = 3.3, p = 0.07
		**Treatment**	**F**_**1,98**_** = 4.8, p = 0.03**
		**Treatment × baseline group**	**F**_**1,98**_** = 4.1, p = 0.04**
Dexamethasone-suppression corticosterone	6 months	Baseline group	F_1,100_ = 0.7, p = 0.4
		**Treatment**	**F**_**1,100**_** = 33.4, p < 0.0001**
		Treatment × baseline group	F_1,100_ = 0.1, p = 0.8
Dexamethasone-suppression corticosterone	12 months	**Baseline group**	**F**_**1,99**_** = 8.9, p = 0.004**
		**Treatment**	**F**_**1,99**_** = 86.7, p < 0.0001**
		**Treatment × baseline group**	**F**_**1,99**_** = 4.3, p = 0.04**
Recovery corticosterone	18 months	**Baseline group**	**F**_**1,98**_** = 11.0, p = 0.001**
		**Treatment**	**F**_**1,98**_** = 13.8, p = 0.0003**
		**Treatment × baseline group**	**F**_**1,98**_** = 5.2, p = 0.03**
ACTH challenge corticosterone	6 months	Baseline group	F_1,100_ = 0.5, p = 0.5
		Treatment	F_1,100_ = 0.3, p = 0.6
		Treatment × baseline group	F_1,100_ = 0.03, p = 0.9
ACTH challenge corticosterone	12 months	Baseline group	F_1,99_ = 0.3, p = 0.6
		Treatment	F_1,100_ = 0.01, p = 0.9
		Treatment × baseline group	F_1,100_ = 0.1, p = 0.7

## Discussion

We manipulated yolk corticosterone levels to explore the effects of prenatal glucocorticoid exposure on the function of the HPA axis throughout offspring lifespan. We found that birds prenatally exposed to elevated embryonic corticosterone took longer to terminate a stress response after the stressor had ended and had reduced negative feedback efficacy after dexamethasone injection. In addition, we found that prenatal exposure to corticosterone resulted in different stress responses depending on the postnatal environment. Specifically, the adult CORT-treated birds displayed two distinct patterns of stress responses that were dependent on their baseline corticosterone concentrations. CORT-treated birds with low adult baseline corticosterone had significantly lower stress-induced corticosterone levels compared to all other birds in the study. These patterns appear to be influenced by the current social conditions faced by the birds, as CORT-treated birds experiencing higher levels of social aggression also had higher baseline corticosterone levels prior to the acute stress test, while those experiencing lower levels of aggression had lower baseline corticosterone prior to the acute stress test. In other words, it appears that in our study the level of social aggression experienced by an individual embryonically exposed to corticosterone influenced their baseline corticosterone levels, and their baseline levels then influenced their stress-induced corticosterone levels.

We first sought to address how embryonic exposure to glucocorticoids, which is an indicator of the maternal environment^22^, alters offspring HPA axis function throughout their lifespan. We therefore conducted a stress series at 6, 12, and 18 months of age to map the long-term effects of prenatal glucocorticoid exposure on offspring response to stress. Compared to control birds, CORT-treated birds were less able to terminate a stress response either following injection with the synthetic glucocorticoid dexamethasone or through natural recovery after the stress had ended. This suggests that prenatal treatment with corticosterone resulted in a change to the negative feedback systems of the HPA axis, since an individual’s ability to turn off a stress response is indicative of the strength of negative feedback in their HPA axis^11,70^. The persistence of this trend of impaired recovery through the 18-month time period, which is approximately three-quarters of the average lifespan of these birds, implies lasting consequences of early-life stress exposure. Previous studies suggest that prenatal stress can be detrimental to offspring, primarily through dysregulation of the HPA axis^6,9,23^. Further, this dysregulation is associated with increased levels of oxidative stress and rates of telomere shortening^16,71,72^, impaired brain growth and cognitive function^73^, and increased disease susceptibility^6,23^. Thus, impaired recovery due to prenatal corticosterone exposure may come at a cost to offspring in terms of their overall health and longevity. Nevertheless, an alternative, but not mutually exclusive, possibility is that this prolonged recovery may be beneficial in a stressful environment, since glucocorticoids are hypothesized to mobilize energy stores that not only help the animal to recover from the current stressor but also prepare them for future stressors^8,74^. Mathematical modeling has tested this prediction and found some support, with the authors predicting that prolonged recoveries are linked to anticipated future conditions^75^. This is intriguing, and one recent study has examined how variability in the environment affects the speed of a single acute stress response^76^, though it did not specifically measure recovery. To our knowledge no study has yet examined how the speed of recovery from one acute stress response may affect subsequent responses to acute stressors in the near future.

Regardless of the consequences of the prolonged recovery period, the underlying cause may be due to changes in glucocorticoid receptors in the birds exposed to embryonic corticosterone. Multiple studies in young rats found that offspring of stressed mothers have reduced expression of both types of glucocorticoid receptors (GRs) in their hippocampi^36,77–79^. Young adult rats with reduced hippocampal GR expression were shown to experience prolonged secretion of corticosterone following a stressor^36,77–79^, suggesting that prenatal corticosterone exposure may affect GR expression. While we did not measure GR expression, we did inject our birds with dexamethasone, which binds to hippocampal GRs and causes inhibition of the HPA axis. Dexamethasone triggered negative feedback, and thus a reduction in corticosterone, in the control birds but not the corticosterone-exposed birds. This supports prenatal corticosterone having an inhibitory effect on GR expression. In addition, while our results are in agreement with these foundational rodent studies, our study is the first to show that these prenatal effects persist throughout the offspring’s lifetime. Importantly, another study in Japanese quail measured relative mRNA expression of GRs in the hippocampus, hypothalamus, and pituitary gland of quail exposed to elevated corticosterone during prenatal development^24^. This well-designed study found that prenatal corticosterone exposure altered GR expression, but interestingly this occurred in a manner that would make negative feedback within the HPA axis more efficient; the opposite of what we report here. A subsequent study observed the same effect across multiple generations of Japanese quail, wherein the offspring of mothers that were prenatally exposed to elevated corticosterone exhibited an attenuated stress response^34^. They again observed changes in GR expression in response to prenatal corticosterone exposure, which suggests the increased negative feedback efficiency observed in the offspring of prenatally stressed mothers is likely mediated by the same mechanism of differential GR expression in the HPA axis^34^. While both of those studies and our study were conducted in Japanese quail, there are some key differences between them that may contribute to our contrasting findings. These differences include, but are not limited to, the concentration of corticosterone injected in the eggs and the day it was injected. While the Zimmer et al. studies^24,34^ injected eggs five days after laying (D5), we chose to inject our eggs the same day they were laid (D0), as previous studies have found that most of the corticosterone deposited into eggs is metabolized by D5^26,27^. Regardless, comparison of these three studies illustrates how different manners of prenatal glucocorticoid exposure can have widely varying postnatal effects.

We also sought to examine how embryonic glucocorticoid exposure interacts with the postnatal environment to affect acute stress responses. While environments are widely variable, we focused specifically on the social environment. Across diverse taxa, a variety of evolutionary pressures caused natural selection to favor sociality, as social groups confer enhanced fitness to all group members^80^. Social groups are commonly structured as a dominance hierarchy based on a ranking system whereby higher-ranked individuals have better access to valuable resources, such as food and mates^80^. Japanese quail form dominance hierarchies through aggressive interactions^81^. While the establishment of hierarchies results in a decrease in aggressive interactions^82^, quail hierarchies are notably unstable and change through time^49^. As a result, social aggression is common in our feral strain of Japanese quail as the dominance hierarchies shift over time^51^. In line with this, we observed social aggression and dominance behaviors at all ages within our population. More specifically, we found that the level of social aggression experienced by individuals embryonically exposed to corticosterone influenced their baseline corticosterone levels, and their baseline levels then influenced their stress-induced corticosterone levels. These findings suggest a causal pathway of effects that were unknown when the study was conceptualized, and were consequently not taken into account in the original study design. To address this, we performed a post-hoc path analysis to evaluate the connections between embryonic corticosterone exposure, experienced aggression, baseline corticosterone, and stress-induced corticosterone. We found that in the presence of embryonic corticosterone exposure, social aggression did relate to baseline corticosterone, which was then related to stress-induced corticosterone levels (Supplementary Fig. S4). We explore the specific connections between these variables below.

Of all the glucocorticoid measures we examined, baseline glucocorticoids are thought to be most sensitive to changes in the environment that impact energy expenditure^83–85^. Both empirical studies in birds^86–88^_,_ as well as mathematical models^89^, have found that baseline corticosterone levels rise in response to frequent environmental challenges. Interestingly, we saw a positive relationship between baseline glucocorticoids and experienced aggression, but only in the CORT-treated birds. As expected in this species, the experienced aggression in individuals from both treatments varied from age to age^49^. Unexpectedly however, high baseline corticosterone concentrations were only positively correlated with high levels of aggression in the CORT-treated birds. Elevating baseline corticosterone levels is thought to prepare animals for energy-demanding activity, like frequent aggressive interactions^90^. Therefore, it is possible that exposure to increased corticosterone levels during embryonic development resulted in programming of the HPA axis to be hypersensitive to stressors later in life^91^. This could lead individuals to secrete higher levels of baseline corticosterone in response to stressful environments in order to better cope with their socially unstable environments^91^.

Both baseline and stress-induced corticosterone levels are often used as measures of organismal fitness^12,13^. While baseline corticosterone provides information on an individual’s energetic state^11^, stress-induced corticosterone provides information on how organisms respond to stress in a given instance^13^. We examined the relationship between baseline and stress-induced corticosterone and found that baseline levels were linked to stress-induced levels, but only in CORT-treated birds. Specifically, we saw two distinct patterns of stress responses that were dependent on baseline corticosterone concentrations. CORT-treated birds with low adult baseline corticosterone had significantly lower stress-induced corticosterone levels compared to control birds. Alternatively, CORT-treated birds with high baseline corticosterone levels had stress-induced corticosterone levels that were higher than their low baseline counterparts, but similar to control birds. This suggests that the CORT-treated birds may have an elevated threshold for the activation of the HPA axis that is somehow linked to the current environment. While the mechanistic underpinnings of this effect remain unclear, the activation of the HPA axis in the birds exposed to embryonic corticosterone appears to be flexible and context-dependent. One possible explanation for this pattern may have to do with the slower recovery from a stress response observed in these birds. Because individuals that are exposed to high levels of maternal glucocorticoids must cope with the negative consequences of a prolonged recovery, their HPA axis may simultaneously be activated less frequently. Our results lend support to this novel prediction, as the CORT-treated birds appear to only strongly activate their HPA axis when baseline corticosterone levels are elevated, possibly due to increased social stress. This effect has consequences for studies investigating the relationship between avian baseline glucocorticoid levels and the plasticity of stress-induced glucocorticoids levels^54^. The relationship between baseline and stress-induced corticosterone levels within a population could depend on the percentage of birds that were prenatally exposed to corticosterone, as our results indicate that birds without experimental prenatal corticosterone exposure may not show a significant relationship between baseline and stress-induced corticosterone while birds experimentally exposed to corticosterone embryonically may show a strong relationship between the two because low-baseline birds fail to mount a strong stress response. Studies often differ on whether or not they find significant correlations between baseline and stress-induced corticosterone^92–96^. Our results demonstrate that prenatal exposure may be a major contributing factor to these different patterns.

Interestingly, despite differences in the stress-induced corticosterone levels of low and high baseline CORT-treated birds, there was not a significant difference between these groups at the sample following injection with dexamethasone at any age. This suggests that baseline corticosterone had no effect on a bird’s ability to turn off the stress response. Additionally, there was no difference in the ability of the CORT-treated birds to respond to the ACTH challenge at 6 and 12 months. This indicates that while the CORT-treated birds with low baseline corticosterone also had low stress-induced corticosterone in response to our bag restraint stressor, they were physiologically capable of releasing corticosterone in response to ACTH. Thus, neither prenatal stress nor baseline corticosterone levels had an effect on the adrenal gland’s capacity to release corticosterone.

## Conclusions and future directions

**Figure 5:**
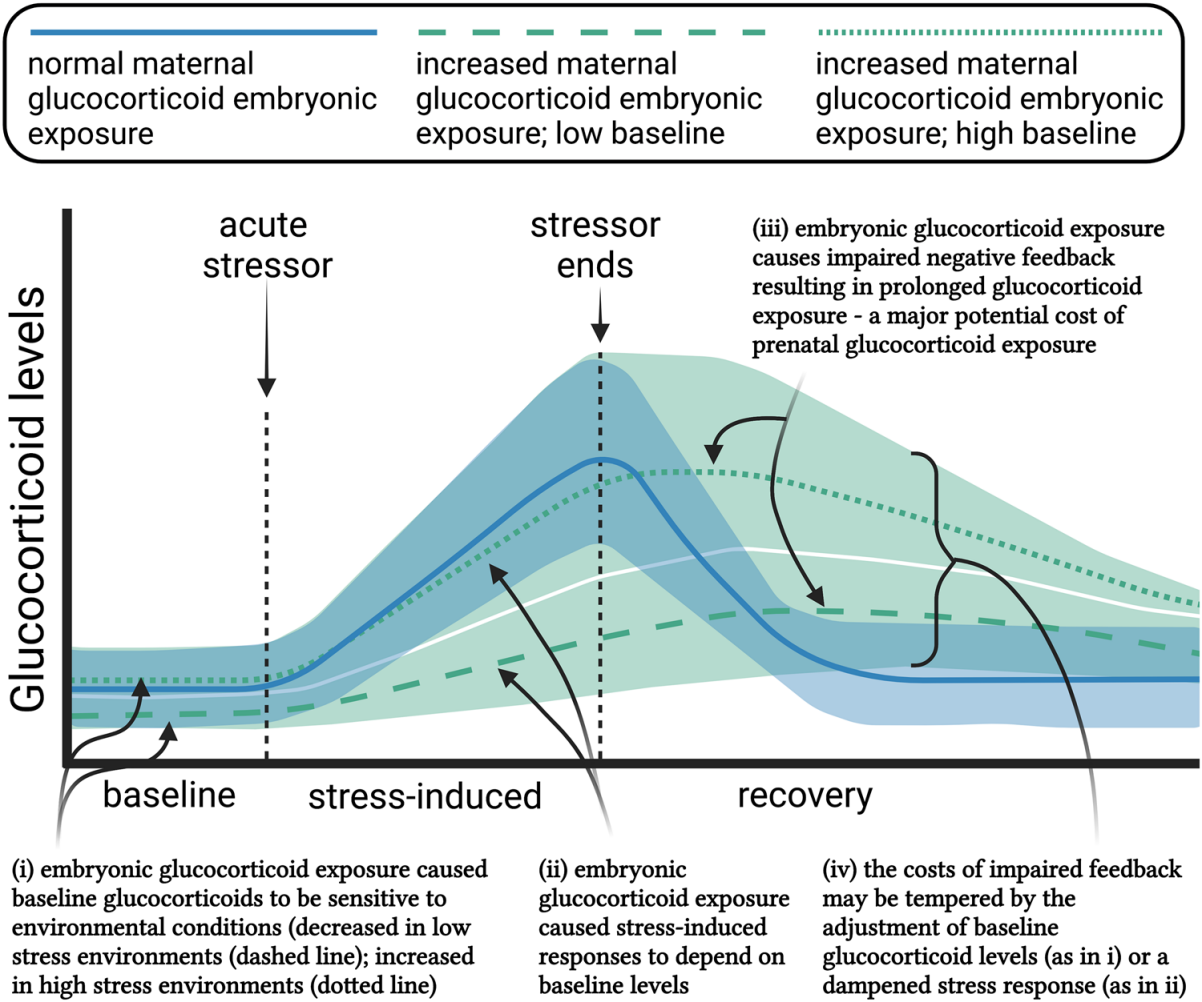
A concept figure detailing how elevated exposure to maternal glucocorticoids during embryonic life could alter the HPA axis and a resulting acute stress response. The mean responses for a normal acute stress response (blue solid line), embryonic glucocorticoid exposure and low postnatal environmental stress (green dashed line), and embryonic glucocorticoid exposure and high postnatal environmental stress (green dotted line) are depicted. The shaded blue (normal) and green (embryonic exposure) areas show ranges of possible acute stress responses based on our data. (i) Baseline glucocorticoids in individuals exposed to elevated embryonic glucocorticoids may be dampened in non-stressful environments (dashed green line), or normal (dotted green line) in a stressful environment (these two groups are separated by a thin white line for clarity). (ii) Upon activation of the HPA axis following a stressor, the magnitude of glucocorticoid increase may depend on baseline glucocorticoid levels. (iii) Regardless of baseline or stress-induced levels, individuals exposed to elevated embryonic glucocorticoids may have impaired negative feedback, resulting in increased cumulative glucocorticoid exposure. (iv) Impaired recovery may have both benefits and consequences to organismal health and fitness. Figure was created with BioRender.com.

An organism’s environment can be unpredictable in terms of weather, social conditions, food availability, and predator attacks. In order to survive, individuals must adjust their physiology and behavior in response to changing environments^29^. Maternal effects are one mechanism to adjust offspring phenotypes for a specific environmental context, as they can have powerful and lasting effects on offspring phenotypes and may even shape them to match prevailing selective conditions^97,98^. Following this, it has been proposed that if a mother’s environment requires a consistently high energetic expenditure, then her elevated glucocorticoid levels can cross the placenta or be deposited in her eggs, where they can then act as a maternal effect to alter the programming of behavioral and physiological pathways of her offspring to potentially increase their fitness^2,4,22,23^. Based on the data presented here, we propose some new wrinkles to this well-worn hypothesis regarding maternal corticosterone exposure in offspring (Fig. 5). Future work should attempt to replicate these findings and test their associated predictions.

Prediction 1: Prenatal glucocorticoid exposure may increase baseline glucocorticoids in postnatal stressful environments (Fig. 5i). Baseline corticosterone levels provide information on an individual’s energetic state^11^, and are strongly influenced by environmental factors^7^. Baseline corticosterone levels are often high in environments where environmental challenges are common^88^. We saw that the CORT-treated birds were able to increase baseline glucocorticoid levels to that of the control birds, but only in environmentally stressful conditions. This suggests that birds prenatally exposed to elevated glucocorticoids may be able to more readily adjust their baseline levels to match environmental conditions. Future work should more thoroughly investigate the magnitude of this effect in a variety of environments, particularly in cases where multiple stressors or repeated stressors are common^29^.

Prediction 2: Activation of an acute stress response may vary depending on prevailing environmental conditions (Fig. 5ii). When facing a stressor, glucocorticoids increase rapidly to mediate the response to and recovery from these challenges^5,74^. Individuals that are unable to elevate glucocorticoids after stress exposure may fail to respond properly^99^. However, exposure to prolonged glucocorticoids can also be costly, as it can impair proper behavioral responses^100^ and cause cellular damage^10,101,102^. Our results suggest that a potential way to balance these costs and benefits is to activate the HPA axis less frequently. Specifically, the CORT-treated birds appear to more strongly activate their HPA axis when baseline corticosterone levels are elevated due to increased environmental stress and to reduce stress-induced levels when environmental stress and baseline glucocorticoids are low. Together, this suggests that a prenatally programmed HPA axis can be activated to various degrees depending on the postnatal environmental conditions and thus displays greater plasticity.

Prediction 3: Prenatal glucocorticoid exposure may impair negative feedback, which could have long-term physiological costs (Fig. 5iii). We saw that CORT-treated birds had impaired negative feedback resulting in prolonged exposure to elevated glucocorticoids at all three ages. However, not all studies find that prenatal glucocorticoid exposure attenuates negative feedback^24,34^, and future work should attempt to pin down how elevated embryonic corticosterone during development results in different effects on the HPA axis. In addition, while a large body of work exists reporting that increased glucocorticoid exposure is linked to a number of costs to offspring health and longevity^6,16,23,72,73,103^, studies specifically investigating how the protracted decline of glucocorticoids after an acute stressor affects health are still needed^104^.

Prediction 4: Impaired negative feedback and prolonged acute stress responses may be beneficial in a stressful environment (Fig. 5iv). Glucocorticoid release during a stressor plays an important role in preparing that animal for future stressors^74^. Thus, even though impaired negative feedback may have consequences, the prolonged recovery may be beneficial in a stressful environment. Because the potential costs of a prolonged recovery are likely to be paid over the long term, future work should explore whether immediate benefits exist which could increase fitness, even if they reduce long-term survival. For example, are individuals with a protracted recovery period following acute stress in a stressful environment better able to respond behaviorally or physiologically to a subsequent stress?

In conclusion, surviving and reproducing in dynamic environments requires phenotypic flexibility. Here, we suggest that maternal glucocorticoids may afford that flexibility in stressful postnatal environments by altering endocrine developmental trajectories. Whether maternal glucocorticoids are ultimately beneficial or detrimental to offspring fitness has long been a subject of debate^2,5,6,9,23,105–107^, and other work provides a framework to design and interpret studies examining the potential adaptive nature of maternal stress^97^. In this study we report that prenatal glucocorticoids appear to have a number of effects on baseline glucocorticoid levels, the degree to which a stress-response is activated, and the speed at which that stress response is terminated. Our data, and predictions that follow those data, indicate that early-life glucocorticoid exposure may facilitate an adaptive stress response based on later-life environmental conditions.

## Supplementary Information

Supplementary Information.

## Data Availability

The datasets generated and analysed during the current study are available from the corresponding author on reasonable request.
