# Supplementary material for: The response to stressors in adulthood depends on the interaction between prenatal exposure to glucocorticoids and environmental context
Source: Sci Rep. 2023 Apr 15;13:6180. doi: 10.1038/s41598-023-33447-x (PMC10105737; doi:10.1038/s41598-023-33447-x)
Supplement: Supplementary file 1 — Supplementary Information. [file 41598_2023_33447_MOESM1_ESM.docx]

**Supplemental Information**

**Title:** The response to stressors in adulthood depends on the interaction between prenatal exposure to glucocorticoids and environmental context.

**Authors:** Ariana D. Majer^1^, Ryan T. Paitz^2^, Gianna M. Tricola^1^, Jack E. Geduldig^1^, Hannah P. Litwa^1^, Jenna L. Farmer^1^, Brenna R. Prevelige^1^, Elyse K. McMahon^1^, Taylor McNeely^1^, Zach R. Sisson^1^, Brian J. Frenz^1^, Alexis D. Ziur^1^, Emily J. Clay^1^, Brad D. Eames^1^, Shannon E. McCollum^1^, and Mark F. Haussmann^1*^

1. Department of Biology, Bucknell University, Lewisburg, PA 17837, USA
2. School of Biological Sciences, Illinois State University, Normal IL 61790, USA

*mark.haussmann@bucknell.edu


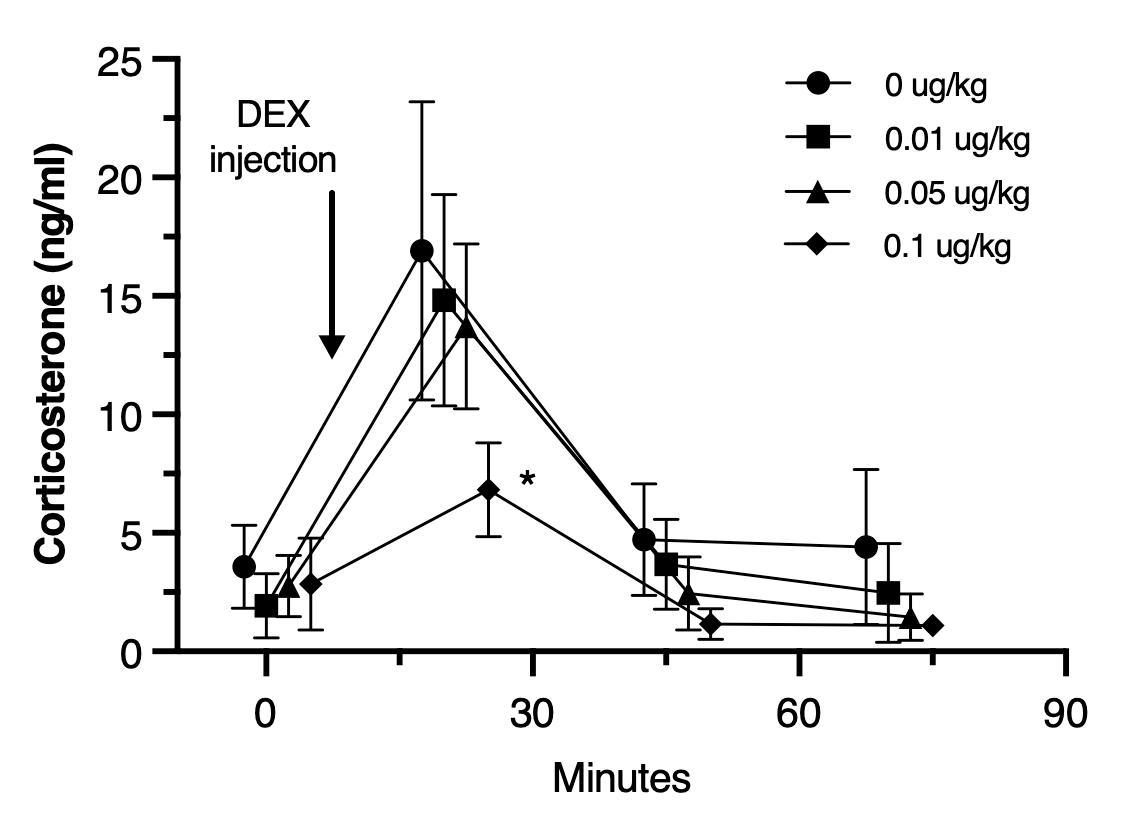


Figure S1. The DEX dose for the study was determined by testing doses at 0 ug/kg (circle), 0.01 ug/kg (square) , 0.05 ug/kg (triangle), 0.1 ug/kg 0 IU/kg (diamond) dissolved in lactated Ringer solution (n = 10 birds for each dose). Initial blood samples pre-injection were taken within approximately 3 minutes of entering the study room. Birds were then injected with one of the DEX doses subcutaneously. After the injection, birds were placed in breathable cloth bags to induce an acute stress response. A second blood sample was taken 20 minutes after the initial disturbance to capture peak plasma CORT concentrations. Afterwards, the birds were removed from the bags and returned to their home pens, to end the stressful stimuli. Final blood samples were taken at 45 minutes and 70 minutes after the initial disturbance to determine the strength of DEX suppression. The 0, 0.01, and 0.05 ug/kg doses did not differ within a time point. Importantly, the 0.1 ug/kg dose had lower CORT values at 20 minutes compared to the other doses, but was also higher than the sample at baseline. This allowed us to examine variation in the negative feedback system to suppress endogenous GC release in response to the acute stressor. Points are jittered for clarity of illustration. Significant differences in the effect of DEX dose at a specific time are marked with an asterisk (Tukey HSD < 0.05).


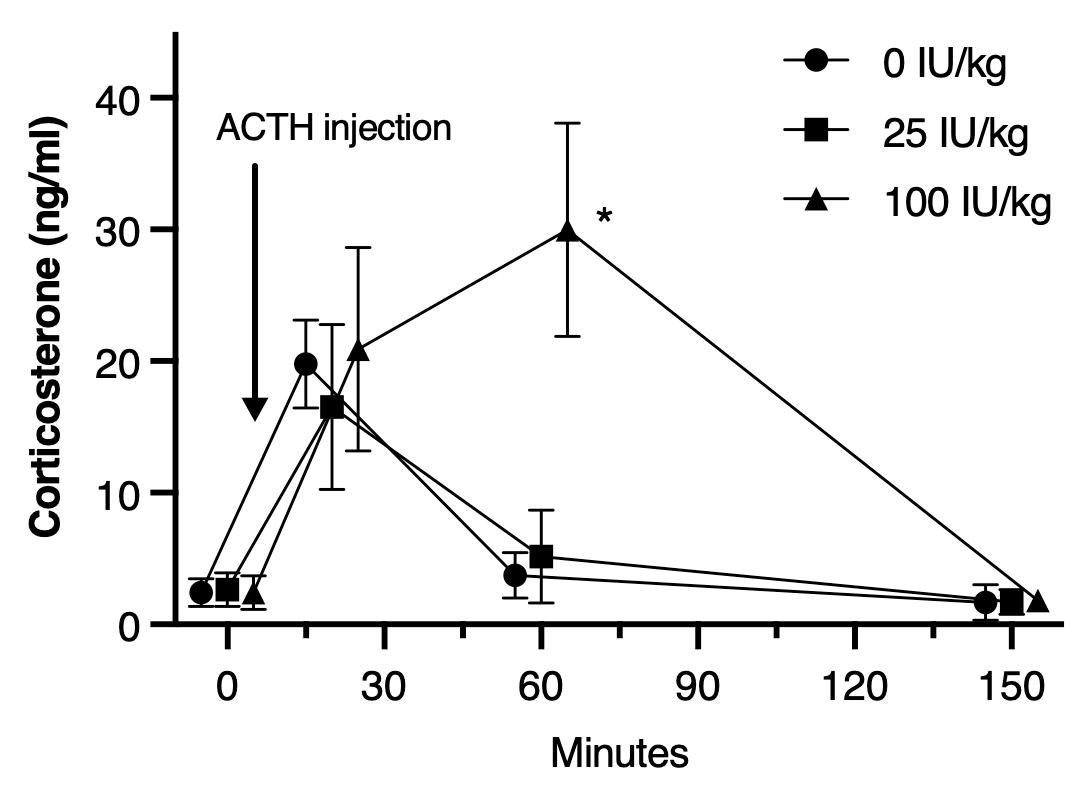


Figure S2. The ACTH dose for the study was determined by testing doses at 0 IU/kg (circle), 25 IU/kg (square), and 100 IU/kg (triangle), dissolved in lactated Ringer solution (n = 10 birds for each dose). Initial blood samples pre-injection were taken within approximately 3 minutes of entering the study room. Birds were then injected with one of the ACTH doses subcutaneously. To assess the ability of ACTH to stimulate adrenal secretion of CORT, birds were placed in breathable cloth bags and a second blood sample was taken 20 minutes after the initial disturbance. After the second blood sample, the birds were removed from the bags and returned to their home pens. Two more blood samples were taken to measure the duration of ACTH effects on CORT at 60 minutes and at 150 minutes. The 0 and 25 iu/kg dose did not differ within a timepoint. We chose to use 100 IU/kg ACTH as the dose in the study because it was effective at producing maximal adrenal stimulation and has been used in previous studies (Rich and Romero, 2005). Points are jittered for clarity of illustration. Significant differences in the effect of ACTH dose at a specific time are marked with an asterisk (Tukey HSD < 0.05).

Table S1. Repeatability of corticosterone concentrations at different timepoints in the HPA challenge. n(i) = number of individuals, n(m) = total number of measurements. Significant p-values are in bold. Bold text shows significant repeatabilities.

| HPA challenge timepoint | Group | *R* | n(i), n(m) | P |
| --- | --- | --- | --- | --- |
| Baseline (3 ages) | All birds | **0.17** | **100, 296** | **0.01** |
|  | Control birds | **0.73** | **50, 147** | **< 0.0001** |
|  | 5ng birds | 0.1 | 50, 149 | 0.14 |
| Stress-induced (3 ages) | All birds | 0.05 | 100, 297 | 0.23 |
|  | Control birds | 0.07 | 50, 147 | 0.36 |
|  | 5ng birds | 0.04 | 50, 149 | 0.57 |
| Dex-suppression (2 ages) | All birds | 0.27 | 100, 199 | 0.09 |
|  | Control birds | 0.16 | 50, 99 | 0.11 |
|  | 5ng birds | 0.04 | 50, 100 | 0.58 |
| ACTH challenge (2 ages) | All birds | 0.11 | 100, 199 | 0.28 |
|  | Control birds | 0.07 | 50, 99 | 0.56 |
|  | 5ng birds | 0.22 | 50, 100 | 0.14 |
| Recovery (2 ages) | All birds | 0.14 | 100, 199 | 0.16 |
|  | Control birds | 0.17 | 50, 99 | 0.24 |
|  | 5ng birds | 0.08 | 50, 100 | 0.58 |

Table S2. Results of generalized linear mixed models (GLMM) on the response to treatment (control or 5ng corticosterone), baseline corticosterone group, and their interaction. Bold values indicate p<0.05.

| Response variable | Predictor variables | Results |
| --- | --- | --- |
| Baseline corticosterone | **Age** | **F_2,197.7_=3.3, p=0.04** |
|  | Treatment | F_1,119.3_=1.3, p=0.3 |
|  | Age x Treatment | F_2,204.2_=0.2, p=0.8 |
| Stress-induced corticosterone | **Age** | **F_2,197.8_=10.4, p<0.0001** |
|  | **Treatment** | **F_1,112.3_=8.0, p=0.006** |
|  | Age x Treatment | F_2,207.3_=0.8, p=0.5 |
| Dexamethasone suppression | Age | F_2,198.2_=2.2, p=0.1 |
|  | **Treatment** | **F_1,111.4_=78.2, p<0.0001** |
|  | Age x Treatment | F_2,208.7_=0.02, p=0.9 |
| ACTH challenge | **Age** | **F_1,99.2_=5.7, p=0.02** |
|  | Treatment | F_1,99.3_=0.001, p=0.9 |
|  | Age x Treatment | F_1,99.2_=0.7, p=0.4 |
| Recovery | **Age** | **F_1,99.0_=6.5, p=0.01** |
|  | Treatment | F_1,99.1_=1.4, p=0.2 |
|  | Age x Treatment | F_1,99.0_=0.2, p=0.6 |

Table S3. Repeatability of experienced aggression across three ages. n(i) = number of individuals, n(m) = total number of measurements. Significant p-values are in bold and bold text shows significant repeatabilities.

| Group | *R* | n(i), n(m) | P |
| --- | --- | --- | --- |
| All birds | 0.24 | 100, 296 | 0.74 |
| Control birds | 0.25 | 50, 147 | 0.59 |
| 5ng birds | 0.30 | 50, 149 | 0.83 |


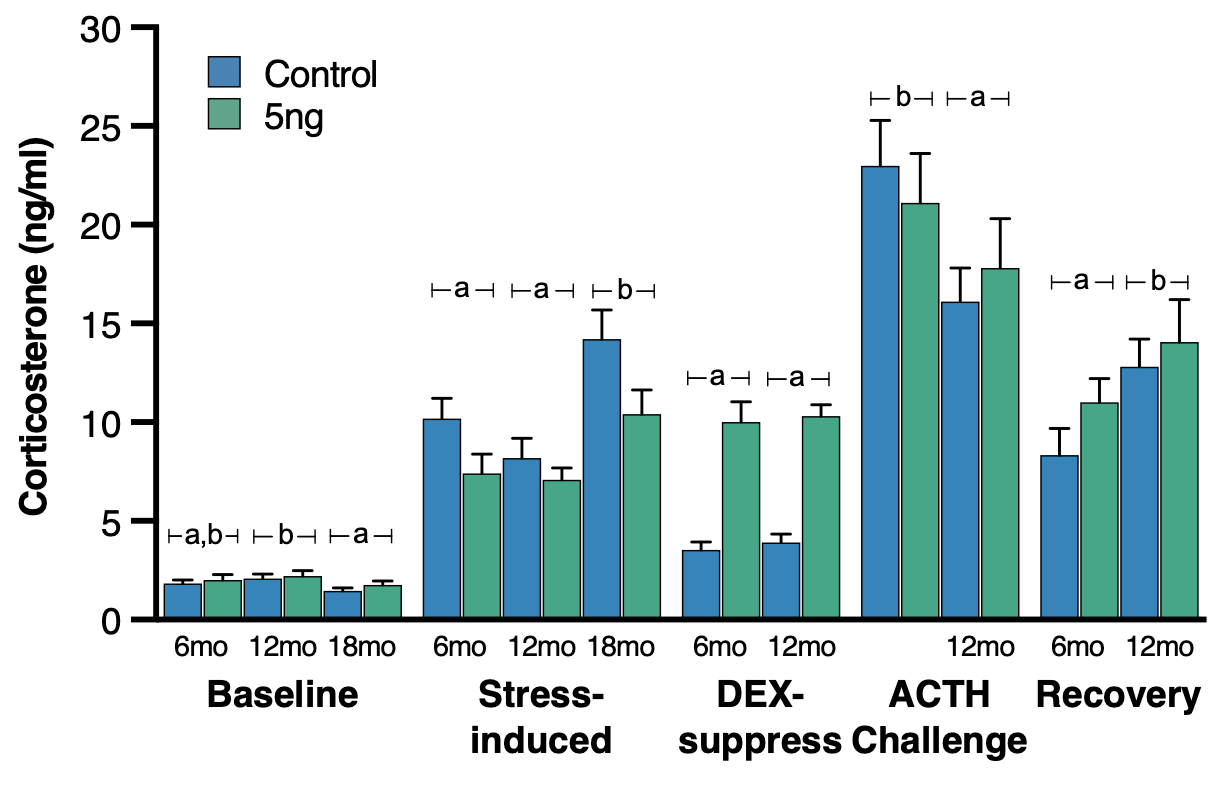


Figure S3. The effect of age (6, 12, and 18 months) and treatment (control in blue; 5ng in green) on baseline, stress-induced, DEX-suppression, ACTH challenge, and recovery corticosterone levels. Mean and standard deviations are plotted. Differences in corticosterone concentrations among ages are denoted by different letters within a stress series timepoint. Differences between treatment groups are not shown.

**Supplementary Note S1: Corticosterone repeatability and age**

A number of studies have explored the repeatability of both baseline and stress-induced corticosterone in individuals. In our study, corticosterone concentrations were repeatable at baseline, but not at any other timepoint (Table S1). This repeatability of baseline corticosterone was driven by the control birds. Interestingly, the corticosterone levels for the embryonic corticosterone birds was not repeatable for any of the HPA challenge timepoints, including baseline (Table S1). The lack of repeatable baseline corticosterone in the embryonic exposed birds is likely due to the experienced aggression, which varied by age, being the driver of baseline glucocorticoids. In addition, the repeatability of baseline corticosterone is currently a topic of debate. While some studies have found baseline corticosterone to be repeatable (Romero & Reed 2008, Guimont & Wynne-Edwards 2006, Narayan et al. 2013), others have found baseline corticosterone to not be repeatable (Romero & Reed 2008, Ouyang et al. 2011). Since baseline corticosterone can be influenced by environmental conditions (Bonier et al. 2009, Romero & Reed 2008), it is possible that these conflicting results may be explained by differences in the stability of the environments of the individuals in the studies.

We also found statistically significant relationships of age on corticosterone across our HPA challenge (Table S2 and Figure S3). Previous studies have reported an increase in baseline corticosterone with age (Meaney et al. 1992). While our data suggests an opposite trend, with baseline levels higher at 12 months than 18 months, this is likely due to the fact that the 12 month timepoint coincided with the breeding season, as previous studies have shown that corticosterone levels are higher during breeding (Love et al. 2004, Moore & Jessop 2003). Both our study and previous studies have also reported higher levels of stress-induced corticosterone in older individuals (Gust et al. 2000), likely due to reduced negative feedback efficacy of glucocorticoids with age (Luisi et al. 1998), which aligns with the glucocorticoid cascade hypothesis (Sapolsky et al. 1986). Additionally, previous studies have noted either a decrease (Ivanisevic-Milovanovic et al. 1998) or no change (Scaccianoce et al. 1995) in responsiveness to ACTH with age, while we also observed a decrease in responsiveness to ACTH with age for all birds. However, it is important to note that we only measured corticosterone levels over the course of one year. One year may not be long enough to observe true age-related changes in the stress response, and more satisfactory conclusions regarding age-related changes in our HPA challenge would require a longer portion of the animal’s lifespan.

References

Bonier F, Martin PR, Moore IT, Wingfield JC. 2009. Do baseline glucocorticoids predict fitness? *Trends Ecol. Evol.* **24**, 634-642.

Guimont FS, Wynne-Edwards KE. 2006. Individual variation in cortisol responses to acute ‘on-back’ restraint in an outbred hamster. *Horm. Behav.* **50**, 252-260.

Gust, D. A. *et al.* 2000. Activity of the Hypothalamic-Pituitary-Adrenal Axis Is Altered by Aging and Exposure to Social Stress in Female Rhesus Monkeys. **85**.

Ivanisevic-Milovanovic, OK, Demjo, M, Loncar-Stevanovic, H, Karakasevic, A, Pantic V. 1998.

Basal and stress induced concentrations of adrenal gland catecholamines and plasma ACTH during aging. Acta Physiol Hung. **85**, 65-75.

Love OP, Breuner CW, Vézina F, Williams TD. 2005. Mediation of a corticosterone-induced reproductive conflict. *Horm. Behav.* **46**, 59-65.

Luisi, S. *et al.* Effect of acute corticotropin releasing factor on pituitary-adrenocortical responsiveness in elderly women and men. 1998. *J Endocrinol Invest* **21**, 449–453.

Meaney MJ, Aitken DH, Sharma S, Viau V. 1992. Basal ACTH, corticosterone and corticosterone-binding globulin levels over the diurnal cycle, and age-related changes in hippocampal type I and type II corticosteroid receptor binding capacity in young and aged, handled and nonhandled rats. *Neuroendo*. **55(2)**, 204-13.

Moore, I. T. & Jessop, T. S. Stress, reproduction, and adrenocortical modulation in amphibians and reptiles. 2003. *Hormones and Behavior* **43**, 39–47.

Narayan EJ, Cockrem JF, Hero J. 2013. Repeatability of baseline corticosterone and short-term corticosterone stress responses, and their correlation with testosterone and body condition in a terrestrial breeding anuran (Platymantis vitiana). *Comp. Biochem. Biophys. A.* **165**, 304-312.

Ouyang JQ, Hau M, Bonier F. 2011. Within seasons and among years: When are corticosterone levels repeatable? *Horm. Behav.* **60**, 559-564.

Romero LM, Reed JM. 2008. Repeatability of baseline corticosterone concentrations. *Gen. Comp. Endocrinol.* **156**, 27-33.

Sapolsky, R.M. Stress, the aging brain, and the mechanisms of neuron death. 1986. *MIT Press*.

Scaccianoce, S., Nicolai, R., Cigliana, G., & Angelucci, L. 1995. Reduced glucocorticoid response to corticotropin secretagogues in the aged Sprague-Dawley rat. *Neuroendocrinology*, **62**(1), 32-38.


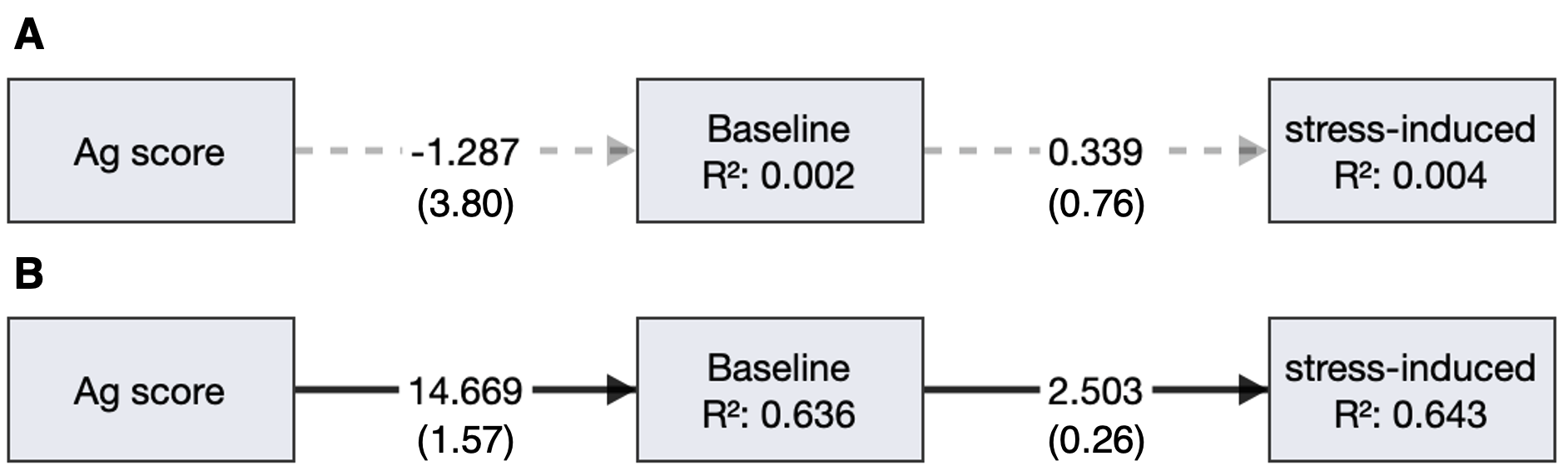


Figure S4. Path coefficients in the hypothesized structural models for (A) control birds, and (B) CORT-treated birds. Numbers are beta coefficients with standard error shown in the parentheses below. R^2^ values are included. Solid arrows show significant relationships, while dashed arrows show nonsignificant relationships. The path analysis was performed in JMP Pro 16.0.0 using the Structural Equation Models – Path analysis platform.
